# Supplementary material for: Rapid isometric hamstring strength scores from professional soccer players at pre-season: Positional differences and relationship to peak force
Source: PLoS One. 2026 Apr 21;21(4):e0347685. doi: 10.1371/journal.pone.0347685 (PMC13098894; doi:10.1371/journal.pone.0347685)
Supplement: Attachment — Submitted filename: Response to reviewers.docx [file pone.0347685.s003.docx]

1. Please ensure that your manuscript meets PLOS ONE's style requirements, including those for file naming. The PLOS ONE style templates can be found at

https://journals.plos.org/plosone/s/file?id=wjVg/PLOSOne_formatting_sample_main_body.pdf and

[https://journals.plos.org/plosone/s/file?id=ba62/PLOSOne_formatting_sample_title_authors_affiliations.pdf](https://eur01.safelinks.protection.outlook.com/?url=https%3A%2F%2Ftrack.editorialmanager.com%2FCL0%2Fhttps%3A%252F%252Fjournals.plos.org%252Fplosone%252Fs%252Ffile%253Fid%3Dba62%252FPLOSOne_formatting_sample_title_authors_affiliations.pdf%2F1%2F010f019cfa3ac2da-0433da2f-a66e-44a2-8be8-e7a95179609d-000000%2FZIk29CmWPoXr-cfuV0v3Oh43OomXCsN-jNsWaorReQY%3D254&data=05%7C02%7Cn.j.ripley%40salford.ac.uk%7C720a7aa5d25f4a58e68608de83e48fd3%7C65b52940f4b641bd833d3033ecbcf6e1%7C0%7C0%7C639093215160947004%7CUnknown%7CTWFpbGZsb3d8eyJFbXB0eU1hcGkiOnRydWUsIlYiOiIwLjAuMDAwMCIsIlAiOiJXaW4zMiIsIkFOIjoiTWFpbCIsIldUIjoyfQ%3D%3D%7C0%7C%7C%7C&sdata=mC3FQ0fg1ZMswIKBAhrIq7xoi3ZuykXZT%2BBOjdII8Zc%3D&reserved=0).

We have addressed the style concerns.

2. We noticed you have some minor occurrence of overlapping text with the following previous publication(s), which needs to be addressed:

- DOI: 10.1519/JSC.0000000000005217

In your revision ensure you cite all your sources (including your own works), and quote or rephrase any duplicated text outside the methods section. Further consideration is dependent on these concerns being addressed

We have attempted to address all aspects identified as part of the review regarding the style and overlapping text. If any specific issues relating to the text can be identified and clarified that would be greatly appreciated.

3. Please include a separate caption for each figure in your manuscript.

We have now included the caption as required.

4. Please include captions for your Supporting Information files at the end of your manuscript, and update any in-text citations to match accordingly. Please see our Supporting Information guidelines for more information: http://journals.plos.org/plosone/s/supporting-information

We have now included the caption as required.

Dear reviewer thank you for your review, we have made all required changes as requested and believe the manuscript is stronger for the review.

Reviewer #1: The article Rapid Isometric Hamstring Strength Scores from Professional Soccer Players at Pre Season: Positional Differences and Relationship to Peak Force presents interesting results, but there are some inconsistences, moreover results, discussion and figures must revised to clarity the manuscript.

Fix the data collection dates, which are currently impossible (listed as 2024–2025). Replace with the accurate preseason-testing window. Line 96: Methods report data collection “between 01/06/2024–08/07/2025,” which spans beyond the present date and likely includes a year mismatch relative to “first week of pre season.” Please confirm the exact dates and season.

We have added the specific dates to the methods section.

L96-98 – “Data collection was across two pre-season period for the 2024-2025 (01/06/2024-4/072024) season and 2025-2026 season (30/06/2025-08/07/2025), with all testing completed between 01/06/2024-08/07/2025.”

Clarify the ANOVA model, the manuscript alternates between “one way” and “two way” ANOVA. Specify the correct factorial structure and apply it consistently. Moreover, to describe limb factor, the author used mixed test, it is necessary to justify the multiplicity strategy across variables/timepoints. The study examines dominant vs non dominant limbs, yet some analyses use paired t tests (for limb differences) and others suggest a two way ANOVA including limb. Please standardize: if limb is a factor, a two way repeated measures (within subject limb) × position (between subject) model may be most appropriate for the variables of interest.

We have clarified the ANOVA model and factorial structure; the Bonferroni correction was applied to account for the municipality. This is more conservative approach than the Sidak correction, highlighting the strength of our findings.

Consider adding SEM to complement CV% to improve applied interpretation.

We agree with the reviewer, and we have added SEM to compliment the reliability, it is worth noting though that this is based on within session data and should be investigated further.

Effect size interpretations must be updated. Clarify the exact factorial structure and ensure the reported η²_p (e.g., 0.17 labeled “small”) is consistent with the correct model and accepted magnitude thresholds. Correct effect size labels: η²ₚ=0.17 is labeled “small” but the manuscript’s own thresholds classify it as large.

Apologies, this was a typo. This has been corrected in the manuscript. It remains a small effect.

Force was sampled at 1,000 Hz and low pass filtered with a 4th order, zero lag Butterworth filter at 50 Hz. It is necessary the justification for 50 Hz cutoff, and the consistent onset threshold application (3 SD).

Please note this is automatic within the proprietary software, we have clarified this within the text.

L252-153 - “automatically applied within the proprietary software for the portable force plates”

Discussion must be rewrite in order to emphasize that positional differences appear only after normalizing rapid force to peak force, and should therefore be interpreted cautiously.

We have re-ordered the paragraphs within the discussion to follow the statistical approach, reliability, positional differences and then discuss the normalization.

Tables
Table 2 header says “absolute and relative values” but only shows relative metrics; align title/content.

Thank you for noting this we have amended this in the table.

Figures:
Provide full, descriptive captions for Figures 1 and 2. Must include posture, goniometer use, hip stabilization, and rejection criteria.

We have included the individual captions, but we have decided not to include the goniometer use, hip stabilization, and rejection criteria as this is described within the text.
